# Are BMI and inflammatory markers independently associated with physical fatigability in old age?

**DOI:** 10.1038/s41366-018-0087-0

**Published:** 2018-05-24

**Authors:** Rachel Cooper, Maria Popham, Adam J Santanasto, Rebecca Hardy, Nancy W Glynn, Diana Kuh

**Affiliations:** 1 MRC Unit for Lifelong Health and Ageing at UCL, 33 Bedford Place, London, WC1B 5JU UK; 2 Department of Epidemiology, University of Pittsburgh, 130 DeSoto Street, Pittsburgh, PA USA

**Keywords:** Epidemiology, Risk factors

## Abstract

**Background:**

Obesity and chronic low-grade inflammation have both been implicated in the onset of physical fatigue. However, few studies have investigated the independence of these associations in older community-dwelling populations. We therefore aimed to investigate the associations of body mass index (BMI) and inflammatory markers at age 60–64 with perceived physical fatigability at age 68 and to assess whether any such associations were independent of each other and potential confounding factors. A secondary aim was to investigate whether any association with BMI extended back into earlier adulthood.

**Methods:**

Participants of the MRC National Survey of Health and Development (*N* = 1580) had BMI and levels of interleukin-6 (IL-6) and C-reactive protein (CRP) measured during clinical assessments at age 60–64. These were related to self-perceived physical fatigability assessed at age 68 using the Pittsburgh Fatigability Scale (PFS) (total score:0 (no physical fatigue)–50 (extreme physical fatigue)).

**Resuts:**

Women had higher mean PFS scores than men (mean (SD): 16.0 (9.1) vs 13.2 (8.9), *p* < 0.01). In sex-adjusted models, BMI, CRP and IL-6 were each associated with PFS scores. When all three factors were included in the same model, BMI and IL-6 remained associated with PFS scores whereas CRP did not. After adjustment for a range of potential confounders, associations of BMI and IL-6 with PFS scores were still evident; fully adjusted differences in mean PFS score = 3.41 (95% CI: 0.59, 6.24) and 1.65 (0.46, 2.84) for underweight and obese participants when compared with normal weight and, 2.78 (1.65, 3.91) when comparing those with an IL-6 of 2.51–8.49 pg/mL with levels <1.50.

**Conclusions:**

BMI and inflammation may both be suitable targets for intervention to reduce the burden of physical fatigability in later life. Further, interventions that target both obesity and elevated levels of IL-6 are likely to be more effective than those focusing on only one.

## Introduction

Physical fatigue is a commonly reported symptom often present even in the absence of clinically manifest disease [1–3]. Levels of fatigue in the general population have been shown to increase with age across adulthood [4–6]. These age-related changes are proposed as key precipitants of declines in activity participation and physical function later in life [1–3, 7]. The importance of fatigue is further highlighted by evidence of longitudinal associations with increased risk of a range of adverse health outcomes including disability and premature mortality [1, 8–12] and by its inclusion, on the basis of such evidence, as a component of geriatric syndromes including frailty [13]. Identifying modifiable risk factors associated with physical fatigue in later life could present new opportunities to reduce its burden in the ageing population.

Chronic low-grade inflammation, a potentially important driver of a wide range of ageing processes [14–16], may be implicated in the onset of physical fatigue [1–3, 17]. Based primarily on findings from animal models and studies of disease-specific patient groups, a number of plausible biological mechanisms have been proposed which could explain a link between inflammation and fatigue [18, 19]. Although generally consistent, epidemiological evidence from general population-based samples is relatively limited as the majority of studies have been small (*n* < 200) and cross-sectional [20–24]. While longitudinal analyses have been undertaken to establish temporality in two larger studies [25, 26], only one of these includes older adults [26].

Adiposity, usually indicated by higher body mass index (BMI), has also been suggested as a potential risk factor for physical fatigue. While this association has been investigated in a number of studies [21–24, 27–34], findings are not fully consistent. Where evidence of association has been found, this is often non-linear with those groups who are underweight and obese reporting greater prevalence of fatigue than those of normal weight [27, 30, 32, 33]. Many studies of this association are limited by cross-sectional designs [28–32] or relatively short follow-up [27]. In addition, although overweight and obesity across life have been related to low-grade inflammation [35–38], few studies have investigated whether the association between BMI and physical fatigue is mediated by, or independent of, inflammation. Establishing this would provide further insight into underlying aetiology and help determine whether one or both sets of factors are meaningful targets for intervention to prevent physical fatigue in older adults.

An additional limitation of existing studies of physical fatigue and, a source of heterogeneity between studies, is the use of a range of different self-reported measures of fatigue none of which account for situation. This is particularly problematic in older populations where adaptations, such as avoiding specific physical tasks or undertaking them more slowly (i.e. self-pacing), may be made to avoid fatigue [39]. If differential levels of adaptation have been taken in different risk profile groups, as suggested by recent findings on obesity history and daily patterns of activity [40], this could introduce bias and attenuate results towards the null. Further research, using whole-body measures of physical fatigability, which assess perceptions of fatigue in the context of specific standard activities of a fixed intensity and duration [3, 39], is thus required.

Using data from a nationally representative birth cohort, the MRC National Survey of Health and Development (NSHD), our primary aim was to investigate the associations of BMI and inflammatory markers at age 60–64 with a validated measure of perceived physical fatigability which takes account of situation at age 68 and to assess whether any such associations were independent of each other and potential confounding factors. A secondary aim was to investigate whether any effect of BMI extended back into earlier adulthood.

## Subjects and methods

The NSHD is a socially stratified sample of 5362 births that occurred during one week of March 1946 in mainland Britain, with regular follow-up across life [41–43]. The 24th data collection was conducted between 2014 and 2015 when study members were asked to complete a postal questionnaire at age 68 and then invited to receive a nurse home visit at age 69 [43]. Of the 2816 study members in the target sample living in England, Scotland and Wales, 2370 (84%) completed the postal questionnaire. In addition, a postal questionnaire was sent to 126 study members living abroad who remain in contact with the study of whom 83 (66%) completed this. No attempt was made to contact the remaining 2420 study members: 957 (18%) had died, 620 (12%) had previously withdrawn from the study, 448 (8%) had emigrated and were no longer in contact with the study, and 395 (7%) had been untraceable for more than 5 years [43].

Ethical approval for the 2014–2015 assessment was obtained from the Queen Square Research Ethics Committee (14/LO/1073) and the Scotland A Research Ethics Committee (14/SS/1009). Study members provided written informed consent.

### Ascertainment of perceived physical fatigability

Physical fatigability was assessed in the postal questionnaire at age 68 using the Pittsburgh Fatigability Scale (PFS) [39]. Study members were asked to indicate the level of physical fatigue they expected or imagined they would feel after completing ten activities on a scale from 0 (no fatigue) to 5 (extreme fatigue). These activities ranged in intensity from low (e.g. watching TV for 2 h) to high (e.g. high-intensity activity for 30 min). Responses to each item were summed to create a total physical fatigability score ranging from 0 (no physical fatigue) to 50 (extreme physical fatigue). Higher physical fatigability was classified as PFS ≥ 15. Where ≤3 items were missing but a related question on whether the activity had been done in the past month was complete (*n* = 289 in analytic sample), values for missing responses were imputed based on the mean value of an individual’s valid responses with adjustments made to take account of the varying intensity levels of the different activities and differences in the levels of fatigue reported by study members who had and had not done each specified activity (see Supplementary text for details).

### Ascertainment of BMI and inflammatory markers

Height and weight measured by nurses using standard protocols at ages 43 and 60–64 were used to calculate BMI (weight (kg)/height ^2^ (m^2^)). BMI was modelled as a continuous variable and also categorised into four standard groups: underweight (<20.0 kg/m^2^); normal weight (20.0–24.9 kg/m^2^); overweight (25.0–29.9 kg/m^2^); obese (≥30.0 kg/m^2^). At age 60–64, measures of body composition were also obtained for 1658 study members who attended a clinical research facility, in the supine position using a QDR 4500 Discovery DXA scanner (Hologic Inc, Bedford, MA). Fat mass index was derived by dividing whole-body fat mass (excluding the head) (kg) by height^2^ (m^2^).

C-reactive protein (CRP) and interleukin-6 (IL-6) were measured in overnight fasting blood samples taken by nurses at age 60–64. After initial processing of the blood samples at clinical research facility laboratories, aliquots were frozen before being transferred to the MRC Human Nutrition Research laboratory in Cambridge where analyses of CRP were processed according to standardised protocols using a particle-enhanced immunoturbidimetric assay. Analyses of IL-6 were subsequently undertaken by the British Heart Foundation Research Centre in Glasgow using ELISA (enzyme-linked immunosorbent assay). Due to the skewed nature of their distributions both CRP and IL-6 were log-transformed when modelled continuously. Both variables were also categorised into four groups; cut-points for CRP were based on CDC/AHA criteria [44] with the addition of an upper category to distinguish acute inflammation as follows: <1.00 mg/L; 1.00–3.00 mg/L; 3.01–10.00 mg/L; >10.00 mg/L. Cut-points for IL-6 were those identified as equivalent to the CRP cut-points in a previous study:[26] <1.50 pg/mL; 1.50–2.50 pg/mL; 2.51–8.49 pg/mL; ≥8.50 pg/mL.

### Covariates

Factors which may confound the main associations of interest were selected a priori. This included: sex; leisure time physical activity and smoking status; symptoms of anxiety and depression; diabetes, cardiovascular disease, respiratory symptoms and medication use; educational level and occupational class. With the exception of the indicators of socioeconomic position, these were assessed at age 60–64.

### Behavioural risk factors

Study members were asked to report whether or not they had participated in any sports, vigorous leisure activities or exercises in their spare time, not including getting to and from work, in the last 4 weeks and, if so, on how many occasions. This information was categorised as: inactive (reported no participation); moderately active (participated in relevant activities 1–4 times in the previous 4 weeks); most active (participated in relevant activities ≥ 5 times in the previous 4 weeks). Self-reported smoking status was categorised as: current; ex; never smoker.

### Health status

The General Health Questionnaire-28 was used to distinguish those study members with symptoms of anxiety and depression from those without using a threshold of ≥5 for caseness [45]. Self-reports of diabetes and doctor diagnosed angina and myocardial infarction from assessments undertaken up to and including age 60–64 were used to distinguish between study members with and without reports of type II diabetes and cardiovascular disease. Respiratory symptoms were assessed using the UK Medical Research Council’s standardised questions [46]. A group with the most severe symptoms were identified who reported one or more of the following: a wheezy or whistling chest most days or nights; usually bringing up phlegm or coughing in the morning or during the day or night in winter for at least 3 months each year; or more than one chest illness in the last 3 years that kept them off work or indoors for 1 week or more. A variable was derived to indicate whether or not study members reported taking medication that may affect their inflammatory status or levels of fatigue. Medications included were: lipid lowering drugs, aspirin, oral steroids, hormone replacement therapy, anti-depressants, hypnotics, anti-inflammatories, disease modifying anti-rheumatic drugs, thyroid medication, weight reduction drugs and central nervous system stimulants.

### Socioeconomic position

Educational level attained by age 26 was categorised into five groups: degree or higher; A levels, usually attained at age 18, or their equivalents; O levels, usually attained at age 16, or their equivalents; CSE, clerical course or equivalent; none. Occupational class at age 53 (or if not available, the most recent measure in adulthood) was categorised using the Registrar General’s Social Classification into three groups: high (I or II); middle (IIINM or IIIM); low (IV or V).

We also investigated the possibility of reverse causality by taking account of prior reports of fatigue using data from the nurse home visit at age 43. Fatigue was ascertained at this age using responses to the question ‘over the last year how often have there been days when you tired out very easily?’ with six response options collapsed into three categories: no (never); occasional (occasionally or sometimes); frequent (quite often, very often or always), consistent with previous NSHD analyses [47].

### Statistical analyses

To address the primary aim, linear regression models were used to test the associations of BMI, IL-6 and CRP at age 60–64 with the PFS score at age 68. Likelihood ratio tests of interactions between sex and each explanatory factor were undertaken and all subsequent models were sex-adjusted as no evidence of sex differences was found. Tests of deviation from linearity were undertaken for each explanatory measure by: adding a quadratic term to a sex-adjusted model including a continuous linear term and; comparing models with the categorised version of each measure treated as categorical and linear. As there was evidence of deviations from linearity for all three main associations (quadratic terms for BMI and IL-6 *p* < 0.01; likelihood ratio tests, *p* < 0.01 for BMI and IL-6 and *p* = 0.04 for CRP) all subsequent models were run using the categorised versions of the explanatory factors. The associations of BMI, IL-6 and CRP with the PFS score were then mutually adjusted before potential confounding factors and fatigue at 43 were added to subsequent models.

To confirm that any association between BMI and PFS was driven by adiposity, analyses were run to test the association of DXA-derived fat mass index with the PFS score in the sub-group of study members with this measure. Analyses of BMI were then repeated using the measure from age 43.

To maintain statistical power and minimise bias, missing values of the covariates in the sample with complete data on BMI and both inflammatory markers at age 60–64 and the PFS score at 68 (*n* = 1580) were imputed (leisure time physical activity (*n* = 36), smoking (*n* = 97), symptoms of anxiety and depression (*n* = 32), diabetes (*n* = 1), cardiovascular disease (*n* = 148), respiratory symptoms (*n* = 141), educational level (*n* = 79), occupational class (*n* = 6) and fatigue at 43 (*n* = 68)) using multiple imputation chained equations implemented in Stata version 14. All analyses were run across 20 imputed data sets and estimates were combined using Rubin’s rules.

Sensitivity analyses were performed to test the main sex-adjusted associations in the: maximum available samples; the sample with complete data; the sample excluding those 289 study members for whom items were imputed when generating the total PFS score.

## Results

Characteristics of the main analytical sample are presented in Table 1. Women had higher mean PFS scores at age 68 than men (*p* < 0.01) and also had greater prevalence of higher perceived physical fatigability (49.4% (women) vs 37.0% (men), *p* < 0.01).

**Table 1:** Characteristics of the MRC National Survey of Health and Development (sample restricted to those with complete data on BMI and inflammatory markers at age 60–64 and perceived physical fatigability at age 68 (maximum *n* = 1580))

	Men		Women	
	*n* ^a^	% or Mean (SD)	*n* ^a^	% or Mean (SD)
Physical Fatigability Scale Score at age 68	760	13.2 (8.9)	820	16.0 (9.1)
BMI at age 60–64^b^				
Underweight	10	1.3	25	3.1
Normal weight	193	25.4	271	33.1
Overweight	367	48.3	305	37.2
Obese	190	25.0	219	26.7
CRP (mg/l) at age 60–64				
<1.00	197	25.9	181	22.1
1.00–3.00	339	44.6	371	45.2
3.01–10.00	185	24.3	224	27.3
>10.00	39	5.1	44	5.4
IL-6 (pg/mL) at age 60–64				
<1.50	281	37.0	319	38.9
1.50–2.50	225	29.6	251	30.6
2.51–8.49	216	28.4	216	26.3
≥8.50	38	5.0	34	4.2
Covariates (at age 60–64 unless indicated otherwise)				
Leisure time physical activity				
Inactive	452	60.9	474	59.1
Moderately active	107	14.4	125	15.6
Most active	183	24.7	203	25.3
Smoking status				
Current	67	9.5	77	9.9
Ex	435	61.5	427	55.0
Never	205	29.0	272	35.1
Symptoms of anxiety & depression				
Yes	87	11.7	165	20.6
No	660	88.4	636	79.4
Type II diabetes				
Yes	60	7.9	34	4.2
No	700	92.1	785	95.9
Cardiovascular disease				
Yes	61	8.9	25	3.3
No	621	91.1	725	96.7
Respiratory symptoms				
Yes	120	17.6	134	17.7
No	561	82.4	624	82.3
Medication use^c^				
Yes	327	43.0^d^	360	43.9^d^
No	433	57.0	460	56.1
Educational level attained by age 26				
None	194	27.1	197	25.1
CSE, clerical course or equivalent	35	4.9	73	9.3
O levels, or their equivalents	111	15.5	225	28.7
A levels, or their equivalents	236	32.9	237	30.2
Degree or higher	141	19.7	52	6.6
Occupational class at age 53				
Low (IV or V)	64	8.5	113	13.8
Middle (IIINM or IIIM)	233	30.9	358	43.7
High (I or II)	458	60.7	348	42.5
Fatigue at age 43	443	61.0	284	36.1
No	443	61.0	284	36.1
Occasional	229	31.5	356	45.3
Frequent	54	7.4	146	18.6

^a^Total *n*s vary due to missing data

^b^Cut-points for BMI (kg/m^2^): underweight (<20.0); normal weight (20.0–24.9); overweight (25.0–29.9); obese (≥30.0)

^c^Prescribed at least one of the following medications: lipid lowering drugs; aspirin; oral steroids; hormone replacement therapy; anti-depressants; hypnotics; anti-inflammatories; disease modifying anti-rheumatic drugs; thyroid medication; weight reduction drugs; central nervous system stimulants

^d^1 medication: men: 24.1%; women: 26.8%; 2 medications: men: 16.5%; women: 11.1%; ≥3 medications: men: 2.5%; women: 6.0%

In sex-adjusted models, BMI, CRP and IL-6 at age 60–64 were each associated with PFS scores at age 68 (Table 2, model 1). Study members who were underweight and those who were obese both had higher mean PFS scores than those who were normal weight; differences in mean PFS score = 4.43 (95% CI:1.37, 7.50) and 4.12 (2.93, 5.30) for the underweight and obese groups when compared with the normal weight group, respectively. For CRP and IL-6, there was evidence of increases in mean PFS scores when comparing those study members in the two middle categories of either measure to those in the lowest category, however, associations were weaker among those with CRP and IL-6 levels >10.00 mg/L and ≥8.50 pg/mL, respectively (Table 2, model 1).

**Table 2:** Associations of BMI, CRP and IL-6 at age 60–64 with Pittsburgh Physical Fatigability Scale (PFS) scores at age 68 (*n* = 1580)

Difference in mean PFS score at age 68 (95% CI)				
Model: Adjustments:	1 Sex	2 1+other main explanatory factors	3 2+covariates	4 3+fatigue at 43
BMI				
Underweight	4.43 (1.37, 7.50)	4.47 (1.45, 7.48)	3.71 (0.86, 6.56)	3.41 (0.59, 6.24)
Normal weight	0	0	0	0
Overweight	1.33 (0.27, 2.39)	0.93 (−0.13, 1.98)	0.39 (−0.62, 1.40)	0.49 (−0.50, 1.49)
Obese	4.12 (2.93, 5.30)	2.87 (1.63, 4.11)	1.64 (0.44, 2.84)	1.65 (0.46, 2.84)
CRP (mg/l)				
<1.00	0	0	0	0
1.00–3.00	0.50 (–0.62, 1.62)	−0.24 (−1.36, 0.87)	−0.09 (−1.14, 0.97)	−0.02 (−1.06, 1.02)
3.01–10.00	2.63 (1.38, 3.89)	0.48 (−0.84, 1.80)	0.64 (−0.62, 1.90)	0.56 (−0.68, 1.81)
>10.00	1.43 (−0.71, 3.57)	−1.28 (−3.53, 0.97)	−1.83 (−3.96, 0.30)	−1.87 (−3.98, 0.23)
IL-6 (pg/mL)				
<1.50	0	0	0	0
1.50–2.50	2.33 (1.27, 3.39)	1.92 (0.83, 3.01)	1.25 (0.22, 2.29)	1.27 (0.24, 2.29)
2.51–8.49	4.76 (3.66, 5.85)	4.04 (2.86, 5.23)	2.74 (1.60, 3.88)	2.78 (1.65, 3.91)
≥8.50	2.09 (−0.07, 4.25)	2.12 (−0.15, 4.39)	1.39 (−0.76, 3.54)	1.56 (−0.57, 3.69)

Model adjustments:

1: Sex (likelihood ratio tests of sex interaction: BMI *p* = 0.09, CRP *p* = 0.27, IL-6 *p* = 0.57)

2: Sex and other two main explanatory factors

3: Model 2 plus behavioural risk factors (leisure time physical activity and smoking status); health status (symptoms of anxiety and depression, type II diabetes, cardiovascular disease, respiratory symptoms, medication use) and; indicators of socioeconomic position (educational level attained, occupational class)

4: Model 3 plus fatigue at age 43

For brevity, results are not presented for each individual set of adjustments. Please see Supplementary table 1 for findings from models including separate adjustments for each group of covariates included in models 2 and 3

Analyses run across 20 imputed data sets and results combined using Rubin’s rules. Cut-points for BMI (kg/m^2^): underweight (<20.0); normal weight (20.0–24.9); overweight (25.0–29.9); obese (≥30.0)

When BMI, IL-6 and CRP were included in the same model, associations of BMI and IL-6 with PFS scores were only partially attenuated (Table 2, model 2). However, the association between CRP and PFS scores was fully attenuated; adjustment for IL-6 had a greater impact on these estimates than adjustment for BMI although both factors contributed to this attenuation (Supplementary table 1). Further attenuation of the associations of BMI and IL-6 with PFS scores occurred after adjustment for potential confounding factors (Table 2, model 3), with adjustment for markers of physical health having the greatest impact (Supplementary table 1). For BMI, these adjustments had a greater impact on the effect sizes for the obese group than for the underweight group. Adjustment for fatigue at 43 had limited impact and associations of BMI and IL-6 with PFS scores were still evident in the fully adjusted model (Table 2, model 4); differences in mean PFS score = 3.41 (0.59, 6.24) and 1.65 (0.46, 2.84) for the underweight and obese groups when compared with the normal weight group and, 2.78 (1.65, 3.91) when comparing those with an IL-6 of 2.51–8.49 pg/mL with those with levels <1.50.

Among the sub-group of study members with DXA measures of fat mass at age 60–64, there was evidence of a linear association between higher fat mass index and higher PFS scores (Table 3). Obesity at age 43 was found to be associated with higher mean PFS scores at age 68; sex-adjusted difference in mean PFS score = 4.91 (3.25, 6.58) when comparing those who were obese at age 43 with those who were normal weight (Table 3). There was borderline evidence that this association was stronger among women than men (differences in mean PFS scores when comparing obese vs normal weight = 2.72 (0.13, 5.31) for men and 6.40 (4.23, 8.57) for women, *p*-value for sex interaction = 0.05). There was no evidence of an association between underweight at this younger age and PFS. As for BMI at age 60–64, associations of fat mass index at age 60–64 and BMI at age 43 with PFS were partially attenuated after adjustments for inflammatory markers and other covariates but both associations were still evident in fully adjusted models.

**Table 3:** Associations of BMI at age 43 and fat mass index at age 60–64 with Pittsburgh Physical Fatigability Scale (PFS) scores at age 68

Difference in mean PFS score at age 68 (95% CI)				
Model: Adjustments:	1 Sex	2 1+other main explanatory factors	3 2+covariates	4 3+fatigue at 43
BMI at age 43 (*N* = 1502)				
Underweight	0.91 (−1.15, 2.97)	0.93 (−1.10, 2.96)	0.66 (−1.27, 2.59)	0.41 (−1.50, 2.31)
Normal weight	0	0	0	0
Overweight	0.23 (−0.78, 1.23)	−0.40 (−1.41, 0.60)	−0.77 (−1.72, 0.19)	−0.69 (−1.64, 0.25)
Obese	4.91 (3.25, 6.58)	3.60 (1.92, 5.27)	2.38 (0.74, 4.02)	2.51 (0.89, 4.13)
Fat mass index at age 60–64 (*N* = 1189)				
Per 1 kg/m^2^	0.77 (0.61, 0.94)	0.65 (0.47, 0.84)	0.46 (0.29, 0.64)	0.46 (0.28, 0.63)

Model adjustments:

1: Sex (likelihood ratio tests of sex interaction: BMI *p* = 0.05, fat mass index *p* = 0.93; quadratic terms: BMI *p* = 0.001, fat mass index *p* = 0.32)

2: Sex, IL-6 and CRP

3: Model 2 plus behavioural risk factors (leisure time physical activity and smoking status); health status (symptoms of anxiety and depression, type II diabetes, cardiovascular disease, respiratory symptoms, medication use) and; indicators of socioeconomic position (educational level attained, occupational class)

4: Model 3 plus fatigue at age 43

Sample restricted to those with complete data on BMI and inflammatory markers at age 60–64 and physical fatigability at age 68 with Ns lower due to missing data on BMI at 43 and fat mass index in the main analytic sample

Analyses run across 20 imputed data sets and results combined using Rubin’s rules. Cut-points for BMI (kg/m^2^): underweight (<20.0); normal weight (20.0–24.9); overweight (25.0–29.9); obese (≥30.0)

Findings were very similar and conclusions remained the same when the main models were rerun on: maximum available samples; the sample with complete data and; the sample excluding those study members with imputed PFS items (Supplementary table 2).

## Discussion

In a nationally representative study population, BMI and IL-6 (but not CRP) at age 60–64 were independently associated with perceived physical fatigability at age 68. Those who were underweight or obese and those with higher levels of IL-6 (up to 8.50 pg/mL) had higher mean levels of perceived physical fatigability than those of normal weight and with lower levels of IL-6, respectively. These associations were not fully explained by adjustment for a range of potential confounding factors including leisure time physical activity, smoking, health status and socioeconomic position. Higher fat mass index at age 60–64 and obesity earlier in adulthood also showed independent associations with perceived physical fatigability at age 68.

Our finding of a non-linear association between BMI at age 60–64 and perceived physical fatigability at age 68 is consistent with findings from some other previous studies [27, 30, 32, 33]. However, as these previous studies were cross-sectional and/or limited to women only, our study of both sexes using a validated measure of situational fatigue which also examines inter-relationships with inflammation provides important new insights.

In one of the only other prospective, population-based studies of the association between inflammation and self-reported fatigue, participants in the Whitehall II study with higher levels of CRP and IL-6 at a mean age of 50 years had greater odds of incident fatigue after 3 years of follow-up than participants with low levels of both markers whereas those participants with higher levels of only one of these two markers did not [26]. When we ran additional analyses to investigate the combined effects of each pair of explanatory factors using a similar method to this previous study to facilitate comparison (Supplementary table 3), the results showed that in our study population, higher levels of IL-6 were associated with higher perceived physical fatigability even if CRP levels were lower.

There is a plausible explanation for our finding. Experimental evidence suggests that inflammation may be related to physical fatigue via the direct influence of circulating cytokines on the central nervous system [48, 49]. It is proposed that a number of symptoms including physical fatigue may be a manifestation of changes in neuronal function that occur as a direct result of this process [18, 19, 50]. If this is one of the explanations for our observed associations, IL-6, a pro-inflammatory cytokine that can cross the blood–brain barrier, would be expected to play a more direct role than CRP, an acute phase reactant, which is further downstream and does not cross this barrier.

That we observe a levelling off in associations among those with inflammatory markers above the 95th percentile suggests that chronic low-grade inflammation, that typifies ageing, rather than acute infection, which results in temporarily elevated levels, is driving the observed associations. Higher BMI across life is associated with greater cumulative exposure to chronic low-grade inflammation [35–38], partly explained by the fact that adipose tissue produces and secretes pro-inflammatory cytokines [35]. The inflammatory processes described above are therefore one plausible explanation for associations between higher BMI and physical fatigability. However, our findings suggest that the association between BMI and physical fatigability is only partially explained by inflammation. Additional explanations thus need to be considered, especially as associations were also robust to adjustment for indicators of physical and mental health and behavioural risk factors including leisure time physical activity.

Perceived physical fatigability may reflect an imbalance between energy availability and demand [51]. As the energetic demands of any given weight-bearing physical task will increase as BMI increases, especially when this is explained by an increase in fat mass as in our study, individuals with higher BMI would be expected to report higher levels of physical fatigue. In previous NSHD analyses, higher BMI and greater length of exposure to obesity were shown to be associated with lower muscle quality at age 60–64 [52]. When completing any specified physical task those with higher BMI will thus also have lower capacity to complete the task, especially if they have been obese for longer, further increasing their risk of experiencing fatigue. Our finding of an association between obesity earlier in adulthood and higher physical fatigability provides support for this explanation. There was also some evidence to suggest this association may be stronger among women than men, possibly due to women’s greater average fat mass for a given BMI.

While associations of underweight with physical fatigability were more robust to adjustment than those for obesity, only a very small group of study members were underweight in our analytic sample at age 60–64 (*n* = 35). Thus the number of cases of high physical fatigability attributable to underweight, if this association was causal, would be much lower than for obesity. Authors of previous studies that have found higher physical fatigability among those who were underweight have suggested that this could be explained by the fact that low BMI often indicates underlying disease processes [27, 30]. This may explain why associations were observed with underweight at age 60–64 but not 43; disease would be expected to become an increasingly important determinant of low BMI at older ages as the burden of disease increases. However, our association between underweight at age 60–64 and physical fatigability was only moderately attenuated after adjustment for health status, though the potential for residual confounding by other diseases and underlying conditions including those not yet clinically manifest is acknowledged.

Our analyses have a number of key strengths including the use of a relatively large general population-based sample with data ascertained prospectively across life. We were also able to take account of a wide range of potential confounders, including symptoms of anxiety and depression and anti-depressant use, the importance of which has been highlighted in a number of recent studies [53–55]. Another strength is the use of a validated measure of perceived physical fatigability [39] which overcomes some important limitations of other self-report fatigue measures.

To be eligible for inclusion in our analyses, study members had to have completed a postal questionnaire at age 68. As high levels of participation have been maintained across life, the sample remains largely representative of the population from which it was drawn [43]. However, study members of lower SEP and poorer health status are more likely to have been lost to follow-up. While multiple imputation was used to maximise the sample size and minimise the level of bias introduced due to missing data on covariates we did exclude those study members who did not have complete data on BMI and inflammation at age 60–64 to ensure the models of our three main explanatory factors were all run on the same *n*. When we compared those 1580 study members included in analyses with those 515 who had a valid PFS score but did not have complete data on BMI and inflammation there were no differences in mean PFS scores (14.6 vs 15.2, *p* = 0.27), behavioural risk factors, CVD, respiratory symptoms, medication use, occupational class or fatigue at 43 but the group excluded did have higher prevalence of obesity, anxiety and depression and diabetes and lower levels of education. While it is possible that bias may have been introduced due to these sample restrictions, when basic sex-adjusted analyses were rerun on the maximum available samples, findings remained the same (Supplementary table 2).

Another potential limitation of our analyses is the use of only two inflammatory markers which had been measured just once, at age 60–64. Alongside which, our selected measure of physical fatigability was only ascertained at age 68. However, there are strengths of using IL-6 if only a limited number of markers are available [56] and we were able to assess the potential for reverse causality by adjusting for an earlier measure of fatigue. While BMI has been measured at multiple ages in the NSHD, we focused on age 60–64 so that we could investigate inter-relationships with inflammation. In additional analyses, in which we examined BMI at age 43, selected because the prevalence of obesity first exceeded 10% in the NSHD at this age [57], associations extending back into earlier adulthood were evident. This suggests the need to investigate the longer term longitudinal inter-relationships of BMI and inflammation with physical fatigability. This will be especially important in cohorts born more recently who have experienced higher prevalence of obesity, and therefore longer term exposure to low-grade systemic inflammation, from younger ages [57].

Our findings suggest that BMI and inflammation are both suitable targets for intervention to reduce the burden of physical fatigability in later life. In addition, our finding that associations of BMI and IL-6 were additive suggest that interventions that target both risk factors may be more effective than those focusing on only one.

## Electronic supplementary material

Supplementary information

## References

[1] Avlund K (2013). Fatigue in older populations. Fatigue Biomed Health Behav.

[2] Alexander NB, Taffet GE, Horne FM, Eldadah BA, Ferrucci L, Nayfield S (2010). Bedside-to-Bench conference: research agenda for idiopathic fatigue and aging. J Am Geriatr Soc.

[3] Eldadah BA (2010). Fatigue and fatigability in older adults. PM R.

[4] Beutel ME, Weidner K, Schwarz R, Brahler E (2004). Age-related complaints in women and their determinants based on a representative community study. Eur J Obstet Gynecol Reprod Biol.

[5] Loge JH, Ekeberg O, Kaasa S (1998). Fatigue in the general Norwegian population: normative data and associations. J Psychosom Res.

[6] Meng H, Hale L, Friedberg F (2010). Prevalence and predictors of fatigue in middle-aged and older adults: evidence from the health and retirement study. J Am Geriatr Soc.

[7] Gill TM, Desai MM, Gahbauer EA, Holford TR, Williams CS (2001). Restricted activity among community-living older persons: incidence, precipitants, and health care utilization. Ann Intern Med.

[8] Avlund K, Pedersen AN, Schroll M (2003). Functional decline from age 80 to 85: influence of preceding changes in tiredness in daily activities. Psychosom Med.

[9] Moreh E, Jacobs JM, Stessman J (2010). Fatigue, function, and mortality in older adults. J Gerontol A Biol Sci Med Sci.

[10] Hardy SE, Studenski SA (2008). Fatigue predicts mortality in older adults. J Am Geriatr Soc.

[11] Simonsick EM, Glynn NW, Jerome GJ, Shardell M, Schrack JA, Ferrucci L (2016). Fatigued, but not frail: perceived fatigability as a marker of impending decline in mobility-intact older adults. J Am Geriatr Soc.

[12] Basu N, Yang X, Luben RN, Whibley D, Macfarlane GJ, Wareham NJ (2016). Fatigue is associated with excess mortality in the general population: results from the EPIC-Norfolk study. BMC Med.

[13] Fried LP, Tangen CM, Walston J, Newman AB, Hirsch C, Gottdiener J (2001). Frailty in older adults: evidence for a phenotype. J Gerontol A Biol Sci Med Sci.

[14] Fougere B, Boulanger E, Nourhashemi F, Guyonnet S, Cesari M (2017). Chronic inflammation: accelerator of biological aging. J Gerontol A Biol Sci Med Sci.

[15] Franceschi C, Campisi J (2014). Chronic inflammation (inflammaging) and its potential contribution to age-associated diseases. J Gerontol A Biol Sci Med Sci.

[16] Franceschi C, Capri M, Monti D, Giunta S, Olivieri F, Sevini F (2007). Inflammaging and anti-inflammaging: a systemic perspective on aging and longevity emerged from studies in humans. Mech Ageing Dev.

[17] Avlund K (2010). Fatigue in older adults: an early indicator of the aging process?. Aging Clin Exp Res.

[18] Capuron L, Miller AH (2011). Immune system to brain signaling: neuropsychopharmacological implications. Pharmacol Ther.

[19] Dantzer R, O’Connor JC, Freund GG, Johnson RW, Kelley KW (2008). From inflammation to sickness and depression: when the immune system subjugates the brain. Nat Rev Neurosci.

[20] Bautmans I, Gorus E, Njemini R, Mets T (2007). Handgrip performance in relation to self-perceived fatigue, physical functioning and circulating IL-6 in elderly persons without inflammation. BMC Geriatr.

[21] Lim W, Hong S, Nelesen R, Dimsdale JE (2005). The association of obesity, cytokine levels, and depressive symptoms with diverse measures of fatigue in healthy subjects. Arch Intern Med.

[22] Silva JP, Pereira DS, Coelho FM, Lustosa LP, Dias JM, Pereira LS (2011). Clinical, functional and inflammatory factors associated with muscle fatigue and self-perceived fatigue in elderly community-dwelling women. Rev Bras Fisioter.

[23] Valentine RJ, McAuley E, Vieira VJ, Baynard T, Hu L, Evans EM (2009). Sex differences in the relationship between obesity, C-reactive protein, physical activity, depression, sleep quality and fatigue in older adults. Brain Behav Immun.

[24] Valentine RJ, Woods JA, McAuley E, Dantzer R, Evans EM (2011). The associations of adiposity, physical activity and inflammation with fatigue in older adults. Brain Behav Immun.

[25] Cho HJ, Seeman TE, Bower JE, Kiefe CI, Irwin MR (2009). Prospective association between C-reactive protein and fatigue in the coronary artery risk development in young adults study. Biol Psychiatry.

[26] Cho HJ, Kivimaki M, Bower JE, Irwin MR (2013). Association of C-reactive protein and interleukin-6 with new-onset fatigue in the Whitehall II prospective cohort study. Psychol Med.

[27] Bultmann U, Kant IJ, Kasl SV, Schroer KA, Swaen GM, van den Brandt PA (2002). Lifestyle factors as risk factors for fatigue and psychological distress in the working population: prospective results from the Maastricht Cohort Study. J Occup Environ Med.

[28] Chen MK (1986). The epidemiology of self-perceived fatigue among adults. Prev Med.

[29] Coakley EH, Kawachi I, Manson JE, Speizer FE, Willet WC, Colditz GA (1998). Lower levels of physical functioning are associated with higher body weight among middle-aged and older women. Int J Obes Relat Metab Disord.

[30] Jing MJ, Wang JJ, Lin WQ, Lei YX, Wang PX (2015). A community-based cross-sectional study of fatigue in middle-aged and elderly women. J Psychosom Res.

[31] Lin WQ, Jing MJ, Tang J, Wang JJ, Zhang HS, Yuan LX (2015). Factors associated with fatigue among men aged 45 and older: A cross-sectional study. Int J Environ Res Public Health.

[32] Resnick HE, Carter EA, Aloia M, Phillips B (2006). Cross-sectional relationship of reported fatigue to obesity, diet, and physical activity: results from the third national health and nutrition examination survey. J Clin Sleep Med.

[33] Theorell-Haglow J, Lindberg E, Janson C (2006). What are the important risk factors for daytime sleepiness and fatigue in women?. Sleep.

[34] Ward-Ritacco CL, Adrian AL, O’Connor PJ, Binkowski JA, Rogers LQ, Johnson MA (2015). Feelings of energy are associated with physical activity and sleep quality, but not adiposity, in middle-aged postmenopausal women. Menopause.

[35] Visser M, Bouter LM, McQuillan GM, Wener MH, Harris TB (1999). Elevated C-reactive protein levels in overweight and obese adults. JAMA.

[36] Murray ET, Hardy R, Hughes A, Wills A, Sattar N, Deanfield J (2015). Overweight across the life course and adipokines, inflammatory and endothelial markers at age 60-64 years: evidence from the 1946 birth cohort. Int J Obes.

[37] Forsythe LK, Wallace JM, Livingstone MB (2008). Obesity and inflammation: the effects of weight loss. Nutr Res Rev.

[38] Tzoulaki I, Jarvelin MR, Hartikainen AL, Leinonen M, Pouta A, Paldanius M (2008). Size at birth, weight gain over the life course, and low-grade inflammation in young adulthood: northern Finland 1966 Birth Cohort study. Eur Heart J.

[39] Glynn NW, Santanasto AJ, Simonsick EM, Boudreau RM, Beach SR, Schulz R (2015). The Pittsburgh Fatigability scale for older adults: development and validation. J Am Geriatr Soc.

[40] Cooper R, Huang L, Hardy R, Crainiceanu A, Harris T, Schrack JA (2017). Obesity history and daily patterns of physical activity at age 60-64 years: Findings from the MRC National Survey of Health and Development. J Gerontol Med Sci.

[41] Wadsworth M, Kuh D, Richards M, Hardy R (2006). Cohort profile: The 1946 National Birth Cohort (MRC National Survey of Health and Development). Int J Epidemiol.

[42] Kuh D, Pierce M, Adams J, Deanfield J, Ekelund U, Friberg P (2011). Cohort profile: updating the cohort profile for the MRC National Survey of Health and Development: a new clinic-based data collection for ageing research. Int J Epidemiol.

[43] Kuh D, Wong A, Shah I, Moore A, Popham M, Curran P (2016). The MRC National Survey of Health and Development reaches age 70: maintaining participation at older ages in a birth cohort study. Eur J Epidemiol.

[44] Pearson TA, Mensah GA, Alexander RW, Anderson JL, Cannon RO, Criqui M (2003). Markers of inflammation and cardiovascular disease: application to clinical and public health practice: a statement for healthcare professionals from the Centers for Disease Control and Prevention and the American Heart Association. Circulation.

[45] Goldberg DP, Hillier VF (1979). A scaled version of the General Health Questionnaire. Psychol Med.

[46] Medical Research Council.. (1976). Questionnaire on Respiratory Symptoms and Instructions for Interviewers..

[47] Manty M, Kuh D, Cooper R (2015). Associations of midlife to late life fatigue with physical performance and strength in early old age: Results from a British prospective cohort study. Psychosom Med.

[48] Banks WA (2005). Blood-brain barrier transport of cytokines: a mechanism for neuropathology. Curr Pharm Des.

[49] Yarlagadda A, Alfson E, Clayton AH (2009). The blood brain barrier and the role of cytokines in neuropsychiatry. Psychiatry.

[50] Dantzer R, Kelley KW (2007). Twenty years of research on cytokine-induced sickness behavior. Brain Behav Immun.

[51] Schrack JA, Simonsick EM, Ferrucci L (2010). The energetic pathway to mobility loss: an emerging new framework for longitudinal studies on aging. J Am Geriatr Soc.

[52] Cooper R, Hardy R, Bann D, Aihie SA, Ward KA, Adams JE (2014). Body mass index from age 15 years onwards and muscle mass, strength, and quality in early old age: findings from the MRC National Survey of Health and Development. J Gerontol A Biol Sci Med Sci.

[53] Brown PJ, Badreddine D, Roose SP, Rutherford B, Ayonayon HN, Yaffe K (2017). Muscle fatigability and depressive symptoms in later life. Int J Geriatr Psychiatry.

[54] Jokela M, Virtanen M, Batty GD, Kivimaki M (2016). Inflammation and specific symptoms of depression. JAMA Psychiatry.

[55] White J, Kivimaki M, Jokela M, Batty GD (2017). Association of inflammation with specific symptoms of depression in a general population of older people: The English Longitudinal Study of Ageing. Brain Behav Immun.

[56] Metti AL, Aizenstein H, Yaffe K, Boudreau RM, Newman A, Launer L (2015). Trajectories of peripheral interleukin-6, structure of the hippocampus, and cognitive impairment over 14 years in older adults. Neurobiol Aging.

[57] Johnson W, Li L, Kuh D, Hardy R (2015). How has the age-related process of overweight or obesity development changed over time? Co-ordinated analyses of individual participant data from five United Kingdom Birth Cohorts. PLoS Med.

