# Supplementary material for: Are BMI and inflammatory markers independently associated with physical fatigability in old age?
Source: Int J Obes (Lond). 2018 May 24;43(4):832–41. doi: 10.1038/s41366-018-0087-0 (PMC6477893; doi:10.1038/s41366-018-0087-0)
Supplement: Supplementary file 1 — Supplementary information [file 41366_2018_87_MOESM1_ESM.docx]

**Supplementary text 1: Imputing missing items for the Pittsburgh Fatigability Scale**

Where ≤3 of the 10 items of the Pittsburgh Physical Fatigability Scale (PFS) were missing but the related question on whether the activity had been done in the past month was complete, we imputed values for missing responses. These values were based on the mean of an individual’s valid responses with adjustments made to take account of: the varying intensity levels of the 10 different activities and; differences in the levels of fatigue reported by participants who had and had not done each specified activity. The algorithm developed by MP, RC, AS and NWG for this uses sample-specific data to compute sex-specific correction factors based on: whether or not the activity was performed in the past month and, the overall sample mean for the missing item.

The **adjusted PFS** is calculated for those participants with 1, 2 or 3 items missing as follows:

$\begin{matrix} sum of \\ observed items \end{matrix}+\left[ \left( \frac{sum of observed items}{10-number of missing items} \right)*\left( number of missing items \right) \right]+\begin{matrix} \\ sum of correction factors \end{matrix}$

Correction factors are calculated for each missing item. These take account of whether or not the activity was undertaken and sex differences in responses, as follows:

- If a male has not reported their fatigue level for a specified activity AND reported that they do **not** do the activity then their correction for that item is:

$$correction= \left[ \begin{matrix} Average fatigue \\ among males who \\ do not do the activity \end{matrix}- \begin{matrix} Average fatigue \\ among males who \\ do the activity \end{matrix} \right]+\left[ \begin{matrix} Average fatigue \\ among males who \\ do not do the activity \end{matrix}- \begin{matrix} Overall mean fatigue level \\ of the 10 items among males \\ who do not do the activity \end{matrix} \right]$$

- If a female has not reported their fatigue level for a specified activity AND reported that they do **not** do the activity then their correction for that item is:

$$correction= \left[ \begin{matrix} Average fatigue \\ among females who \\ do not do the activity \end{matrix}- \begin{matrix} Average fatigue \\ among females who \\ do the activity \end{matrix} \right]+\left[ \begin{matrix} Average fatigue \\ among females who \\ do not do the activity \end{matrix}- \begin{matrix} Overall mean fatigue level \\ of the 10 items among females \\ who do not do the activity \end{matrix} \right]$$

- If a male has not reported their fatigue level for a specified activity AND reported that they do the activity then their correction for that item is:

$$correction=\left[ \begin{matrix} Average fatigue \\ among males who \\ do the activity \end{matrix}- \begin{matrix} Overall mean fatigue level \\ of the 10 items among males \\ who do the activity \end{matrix} \right]$$

- If a female has not reported their fatigue level for a specified activity AND reported that they do the activity then their correction for that item is:

$$correction=\left[ \begin{matrix} Average fatigue \\ among females who \\ do the activity \end{matrix}- \begin{matrix} Overall mean fatigue level \\ of the 10 items among females \\ who do the activity \end{matrix} \right]$$

As correction factors can be negative, where the total adjusted PFS was negative this was recoded to 0. For a copy of the SAS or STATA code, please contact Nancy W. Glynn at epidnwg@pitt.edu

**Assessment of the adjusted PFS**

Participants from two registries at the University of Pittsburgh were asked to complete the Pittsburgh Fatigability Scale twice within a short timeframe.^1^ The majority of participants completed all items in the PFS on both occasions however, some participants returned incomplete responses to the first mailing. The availability of these two sets of responses enabled a comparison of participants’ PFS scores to assess the outlined method of imputing missing values.

For those participants with ≤3 items missing who had responded to the related question on whether the activity had been done in the past month at mailing 1, adjusted PFS scores were calculated using the imputation algorithm above. Two sets of comparisons were then undertaken by NWG, AS, Robert Boudreau and Megan Marron at the University of Pittsburgh:

1) Using data from the first mailing, the average PFS scores in the sample with complete data (N=681) was compared with average PFS scores in the sample which also included those who had an adjusted PFS score (N=681+112=793). Results showed that inclusion of those with adjusted scores did not alter the distribution of the PFS (Table A).

**Table A:** PFS scores from mailing 1 among sample with complete data only and among sample that also includes those with adjusted scores

|  | N | Mean (SD) | Median (IQR) | n (%) with adjusted  PFS≥15 |
| --- | --- | --- | --- | --- |
| **Complete data:** |  |  |  |  |
| All participants | 681 | 16.0 (9.3) | 15.0 (9.00, 22.0) | 362 (53.2%) |
| Males | 290 | 14.0 (9.0) | 12.0 (7.00, 20.0) | 127 (43.8%) |
| Females | 391 | 17.6 (9.3) | 17.0 (11.0, 24.0) | 235 (60.1%) |
| **Complete data or adjusted PFS score:** |  |  |  |  |
| All participants | 793 | 16.0 (9.3) | 15.0 (8.65, 22.0) | 412 (52.0%) |
| Males | 337 | 14.0 (8.9) | 12.0 (7.00, 20.0) | 145 (43.0%) |
| Females | 456 | 17.4 (9.4) | 16.0 (10.0, 24.0) | 267 (58.6%) |

2) The PFS scores from the two mailings were then compared in: the sample with an adjusted score at the first mailing who had provided a complete response to the second mailing within 30 days (N=77); the sample who had provided complete responses to both mailings within 30 days (N=364). Differences between the PFS scores among the sample with adjusted PFS scores at mailing 1 and complete PFS scores at mailing 2 were minimal and were marginally smaller than those among the sample with complete PFS scores at both mailings (Table B).

**Table B:** Comparison of PFS scores from mailings 1 and 2 (completed within 30 days) among sample with: 1) adjusted PFS scores at mailing 1 and complete PFS scores at mailing 2; 2) complete PFS scores at both mailings

|  | Mean (SD) Median  Range  n (%) PFS≥15 | | |
| --- | --- | --- | --- |
|  | PFS from  1^st^ mailing | PFS from  2^nd^ mailing | Difference in PFS:  2^nd^ mailing – 1^st^ mailing |
| **Adjusted PFS at 1^st^ mailing AND complete PFS at 2^nd^ mailing:** |  |  |  |
| All participants (n=77) | 14.4 (8.2) Med=13.7 Range: 0, 33.1  32 (41.6%) | 15.7 (9.3) Med=14 Range: 0, 41  37 (48.1%) | 1.3 (5.6) Med=0.5  Range: -13.01, 13.7  5 (6.5%) |
|  |  |  |  |
| Males (n=35) | 13.1 (8.0) Med=11.9 Range: 0, 31.4  12 (34.3%) | 14.2 (8.0) Med=14 Range: 0, 36  15 (42.9%) | 1.1 (5.7) Med=0.2  Range: -9.21, 13.7  3 (8.6%) |
|  |  |  |  |
| Females (n=42) | 15.5 (8.3) Med=14.7 Range: 0, 33.1  20 (47.6%) | 17.0 (10.2) Med=17.5 Range: 2, 41  22 (52.4%) | 1.5 (5.6) Med=1.4  Range: -13.01, 12.6  2 (4.8%) |
| **Complete PFS at both mailings:**  All participants (n=364) | 15.8 (9.5) Med=15 Range: 0, 45  189 (51.9%) | 17.7 (9.4) Med=18 Range: 0, 46  220 (60.4%) | 1.8 (4.6) Med=2.0  Range: -14, 17  31 (8.5%) |
|  |  |  |  |
| Males (n=156) | 14.9 (9.8) Med=13 Range: 0, 42  75 (48.1%) | 16.4 (9.3) Med=16 Range: 0, 46  87 (55.8%) | 1.5 (4.8) Med=1.0  Range: -8, 17  12 (7.7%) |
|  |  |  |  |
| Females (n=208) | 16.5 (9.2) Med=16 Range: 0, 45  114 (54.8%) | 18.6 (9.4) Med=19 Range: 0, 44  133 (63.9%) | 2.0 (4.5) Med=2  Range: -14, 15  19 (9.1%) |

(1) Glynn NW, Santanasto AJ, Simonsick EM, Boudreau RM, Beach SR, Schulz R et al. The Pittsburgh Fatigability scale for older adults: development and validation. *J Am Geriatr Soc* 2015; 63(1):130-135.

**Supplementary Table 1: Associations of BMI, CRP and IL-6 at age 60-64 with Pittsburgh Physical Fatigability Scale (PFS) scores at age 68 with separate adjustments for different groups of covariates (N=1580)**

|  | **Difference in mean PFS score at age 68 (95% CI)** | | | | | | | |
| --- | --- | --- | --- | --- | --- | --- | --- | --- |
| **Model adjusted for sex and:** | **-** | **BMI** | **CRP** | **IL-6** | **behavioural risk factors** | **mental health** | **physical health** | **socioeconomic position** |
| **BMI**  Underweight  Normal weight  Overweight  Obese | 4.43 (1.37, 7.50)  0  1.33 (0.27, 2.39)  4.12 (2.93, 5.30) | n/a | 4.41 (1.35, 7.46)  0  1.14 (0.08, 2.21)  3.65 (2.41, 4.88) | 4.49 (1.48, 7.51)  0  0.96 (-0.09, 2.01)  2.95 (1.74, 4.17) | 4.34 (1.34, 7.34)  0  1.08 (0.04, 2.13)  3.77 (2.60, 4.93) | 4.15 (1.14, 7.16)  0  1.37 (0.33, 2.42)  3.98 (2.82, 5.15) | 3.90 (0.94, 6.85)  0  0.75 (-0.28, 1.78)  2.75 (1.57, 3.92) | 4.44 (1.40, 7.48)  0  1.21 (0.15, 2.27)  3.85 (2.67, 5.04) |
| **CRP (mg/l)**  <1.00  1.00 – 3.00  3.01 – 10.00  >10.00 | 0  0.50 (-0.62, 1.62)  2.63 (1.38, 3.89)  1.43 (-0.71, 3.57) | 0  0.20 (-0.91, 1.32)  1.68 (0.39, 2.97)  0.48 (-1.66, 2.62) | n/a | 0  -0.09 (-1.20, 1.03)  1.04 (-0.26, 2.35)  -0.74 (-2.99, 1.52) | 0  0.35 (-0.75, 1.45)  2.23 (0.99, 3.47)  0.90 (-1.20, 3.00) | 0  0.77 (-0.33, 1.87)  2.97 (1.73, 4.20)  1.46 (-0.63, 3.56) | 0  0.27 (-0.81, 1.35)  1.90 (0.69, 3.12)  -0.02 (-2.09, 2.05) | 0  0.42 (-0.69, 1.54)  2.34 (1.08, 3.59)  1.10 (-1.03, 3.23) |
| **IL-6 (pg/ml)**  <1.50  1.50 – 2.50  2.51 – 8.49  ≥ 8.50 | 0  2.33 (1.27, 3.39)  4.76 (3.66, 5.85)  2.09 (-0.07, 4.25) | 0  1.95 (0.88, 3.03)  4.06 (2.94, 5.19)  1.80 (-0.35, 3.95) | 0  2.18 (1.10, 3.27)  4.52 (3.35, 5.70)  2.19 (-0.10, 4.47) | n/a | 0  2.01 (0.96, 3.07)  4.16 (3.07, 5.26)  1.65 (-0.49, 3.79) | 0  2.36 (1.31, 3.40)  4.62 (3.54, 5.69)  2.28 (0.16, 4.41) | 0  1.68 (0.65, 2.72)  3.53 (2.45, 4.61)  1.26 (-0.84, 3.35) | 0  2.21 (1.15, 3.27)  4.53 (3.44, 5.62)  1.73 (-0.43, 3.88) |

Note: Behavioural risk factors (leisure time physical activity and smoking status); mental health (symptoms of anxiety and depression); physical health (type II diabetes, cardiovascular disease, respiratory symptoms, medication use) and; socioeconomic position (educational level attained, occupational class)

Analyses run across 20 imputed datasets and results combined using Rubin’s rules

BMI: body mass index

CRP: C-reactive protein

IL-6: Interleukin-6

Cut-points for BMI (kg/m^2^): underweight (<20.0); normal weight (20.0-24.9); overweight (25.0-29.9); obese (≥30.0)

**Supplementary Table 2: Sex-adjusted associations of BMI, CRP and IL-6 at age 60-64 with Pittsburgh Physical Fatigability Scale (PFS) scores at age 68 using different analytic samples**

|  | **Difference in mean PFS score at age 68 (95% CI)** | | | |
| --- | --- | --- | --- | --- |
|  | **Main analytic sample (N=1580)** | **Maximum available samples** | **Sample with complete data (N=1250)** | **Exclusion of those with imputed PFS scores (N=1291)** |
| **BMI**  Underweight  Normal weight  Overweight  Obese | 4.43 (1.37, 7.50)  0  1.33 (0.27, 2.39)  4.12 (2.93, 5.30) | N=1706  4.56 (1.66, 7.47)  0  1.35 (0.32, 2.38)  4.16 (3.02, 5.30) | 5.58 (2.21, 8.96)  0  1.22 (0.04, 2.40)  4.10 (2.79, 5.41) | 4.40 (1.15, 7.65)  0  1.39 (0.22, 2.55)  3.95 (2.64, 5.26) |
|  |  |  |  |  |
| **CRP (mg/l)**  <1.00  1.00 – 3.00  3.01 – 10.00  >10.00 | 0  0.50 (-0.62, 1.62)  2.63 (1.38, 3.89)  1.43 (-0.71, 3.57) | N=1591  0  0.46 (-0.66, 1.58)  2.72 (1.47, 3.97)  1.51 (-0.62, 3.63) | 0  0.84 (-0.40, 2.07)  2.69 (1.29, 4.10)  2.46 (0.09, 4.83) | 0  0.25 (-0.98, 1.49)  2.06 (0.68, 3.44)  1.41 (-0.91, 3.74) |
|  |  |  |  |  |
| **IL-6 (pg/ml)**  <1.50  1.50 – 2.50  2.51 – 8.49  ≥ 8.50 | 0  2.33 (1.27, 3.39)  4.76 (3.66, 5.85)  2.09 (-0.07, 4.25) | N=1588  0  2.32 (1.26, 3.38)  4.79 (3.70, 5.89)  1.85 (-0.27, 3.98) | 0  1.97 (0.78, 3.16)  4.77 (3.57, 5.98)  2.64 (0.22, 5.06) | 0  2.13 (0.95, 3.31)  4.66 (3.45, 5.86)  2.45 (0.07, 4.84) |

BMI: body mass index

CRP: C-reactive protein

IL-6: Interleukin-6

Cut-points for BMI (kg/m^2^): underweight (<20.0); normal weight (20.0-24.9); overweight (25.0-29.9); obese (≥30.0)

**Supplementary Table 3: Combined associations of BMI, CRP and IL-6 at age 60-64 with Pittsburgh Physical Fatigability Scale (PFS) scores at age 68 (N=1580)**

|  | **Difference in mean PFS score at age 68 (95% CI)** | |
| --- | --- | --- |
|  | **Sex-adjusted** | **Adjusted for sex and other covariates** |
| **BMI-CRP**  low-low  high-low  low-high  high-high | 0  2.64 (1.27, 4.00)  1.28 (0.09, 2.47)  4.41 (3.06, 5.76) | 0  1.29 (-0.01, 2.58)  0.60 (-0.53, 1.74)  2.88 (1.59, 4.18) |
| **BMI-IL-6**  low-low  high-low  low-high  high-high | 0  2.86 (1.53, 4.19)  3.04 (1.88, 4.19)  5.23 (3.87, 6.59) | 0  1.75 (0.48, 3.02)  1.94 (0.84, 3.05)  3.27 (1.95, 4.59) |
| **CRP-IL-6**  low-low  high-low  low-high  high-high | 0  0.98 (-0.32, 2.28)  2.81 (1.54, 4.09)  4.22 (3.00, 5.45) | 0  0.73 (-0.51, 1.96)  1.84 (0.63, 3.04)  2.65 (1.46, 3.84) |

BMI: body mass index

CRP: C-reactive protein

IL-6: Interleukin-6

Low vs high defined as: <30 vs ≥30kg/m^2^ for BMI; ≤3.00 vs >3.00mg/l for CRP; ≤2.50 vs >2.50pg/ml for IL-6

Model adjustments:

1: sex (likelihood ratio tests of sex interaction:, BMI and CRP p=0.09, BMI and IL-6 p=0.27, IL-6 and CRP p=0.32)

2: sex, behavioural risk factors (leisure time physical activity and smoking status); health status (symptoms of anxiety and depression, type II diabetes, cardiovascular disease, respiratory symptoms, medication use) and; indicators of socioeconomic position (educational level attained, occupational class)

Analyses run across 20 imputed datasets and results combined using Rubin’s rules

p-values from formal comparisons of categories in sex-adjusted models –

BMI-CRP: high-low vs low-high p=0.09; high-low vs high-high, p=0.04; low-high vs high-high, p<0.01

BMI-IL-6: high-low vs low-high, p=0.82; high-low vs high-high, p<0.01; low-high vs high-high, p<0.01

CRP-IL-6: high-low vs low-high, p=0.03; high-low vs high-high, p<0.01; low-high vs high-high, p=0.08

p>0.5 for tests of interaction between each pairing of binary variables
